# Dr. William L. Parmenter

**Published:** 1915-10

**Authors:** 


**Dr. William L. Parmenter,** Homoeopathic of Cleveland, 1872, died after a few days' illness at Port Stanley, Ont., September 16, aged 81. The funeral was held from his late residence in Buffalo, September 19. He was born in Gananoque, Ont., where he spent his boyhood. He graduated at Kingston and assisted in the first survey of Canadian railroads, before engaging in the study of medicine. He was a practitioner of Buffalo from 1872 until a few years ago, when he moved to Port Stanley but spent much of his time in Buffalo. He is survived by two sons, Dr. John Parmenter, formerly of Buffalo, but for several years residing at Geneva; and Dr. Frederick J. Parmenter of Buffalo.

